# A chemical biology screen reveals a role for Rab21-mediated control of actomyosin contractility in fibroblast-driven cancer invasion

**DOI:** 10.1038/sj.bjc.6605469

**Published:** 2009-12-01

**Authors:** S Hooper, C Gaggioli, E Sahai

**Affiliations:** 1 Tumour Cell Biology Laboratory, Cancer Research UK London Research Institute, 44 Lincoln's Inn Fields, London WC2A 3PX, UK; 2 INSERM UNITE U634, Nice, France

**Keywords:** actomyosin contractility, cancer invasion, stromal fibroblast, matrix remodelling, Rab

## Abstract

**Background:**

Carcinoma-associated fibroblasts (CAFs) can promote the progression of tumours in many ways. They can remodel the extracellular matrix to generate an environment that enables the invasion of cancer cells. We hypothesised that compounds that prevent matrix remodelling by CAFs would block their ability to promote carcinoma cell invasion.

**Methods:**

We designed a screen for compounds that interfere with CAF-promoted matrix remodelling. Hits from this screen were investigated in organotypic invasion models of squamous cell carcinoma (SCC).

**Results:**

We find that lovastatin and simvastatin reduce matrix remodelling by fibroblasts and thereby reduce SCC invasion. This class of compounds exert their effects partly through disrupting the function of Rab proteins, and we show a new role for Rab21 in promoting cancer cell invasion promoted by CAFs.

**Conclusions:**

Rab21 is required for CAFs to promote the invasion of cancer cells. It enables the accumulation of integrin *α*5 at the plasma membrane and subsequent force-mediated matrix remodelling.

The local invasion of cancer is a major problem and is frequently associated with distant metastases. However, cancer cell invasion is not solely determined by the characteristics of the cancer cells but also by multiple cell types within tumours, including fibroblasts and a range of immune cells (Egeblad *et al*, 2005). Matrix remodelling by fibroblasts generates paths through the extracellular matrix (ECM) that are subsequently used by cancer cells (Gaggioli *et al*, 2007). This process requires MMPs, integrins and the actin cytoskeleton (Friedl and Wolf, 2008). In particular, coordination of integrin-mediated matrix attachment and actomyosin force generation enable fibroblasts to move matrix components such as collagen fibres (Grinnell, 2003; Meshel *et al*, 2005). This process is regulated at many levels including the delivery of integrins to the plasma membrane, integrin activation, actin polymerisation and regulation of myosin activity (Grinnell, 2003). Integrin delivery and recycling is mediated through various vesicular compartments and is largely controlled by Rab family small G proteins (Jones *et al*, 2006; Pellinen and Ivaska, 2006). Both Rab 5 and Rab 21 have been localised to early endosomes from which integrins can be recycled back to the plasma membrane by either Rab4- or Rab11-dependent route (Jones *et al*, 2006; Pellinen *et al*, 2006). Ligand-bound integrins initiate a range of intracellular signalling pathways, including activation of actomysoin contraction. Myosin activity is largely controlled by the phosphorylation of MLC, which can be performed by numerous kinases including the RhoA-regulated kinases ROCK1 and ROCK2 (Olson and Sahai, 2009). Both ROCK and MLC function are required for matrix remodelling by fibroblasts (Gaggioli *et al*, 2007; Honjo *et al*, 2007; Yuge *et al*, 2007).

At a microscopic level matrix remodelling can be visualised by the appearance of holes in the ECM adjacent to the carcinoma-associated fibroblasts (CAFs) and the concentration of matrix fibres around cells. Many fibroblasts also have the ability to contract collagen gels at the macroscopic level (Ngo *et al*, 2006). We noted a good correlation between matrix remodelling at the microscopic and macroscopic levels and with the ability of CAFs to promote the invasion of squamous cell carcinoma (SCC) cells. We therefore hypothesised that matrix contraction by CAFs could be used as a screening method to identify compounds that prevent matrix remodelling and hence would reduce fibroblast-stimulated carcinoma cell invasion.

In this work we screen almost 200 compounds for their effect on matrix remodelling by CAFs. We find that two HMG-CoA reductase inhibitors reduce matrix remodelling by fibroblasts and consequently diminish cancer invasion. This class of compounds exert their effects partly through disrupting the function of Rab proteins and we show a new role for Rab21 in matrix remodelling associated with delivery of integrin *α*5 to the plasma membrane and the generation of contractile force.

## Materials and methods

### Cell culture and transfection

Carcinoma-associated fibroblasts and SCC12 were cultured as described by Gaggioli *et al* (2007). HN-CAF were transfected with 100 nM siRNA using Dharmafect 1 (Dharmacon; catalogue no. T-2001-03) 2 *μ*l per well in six-well plates, Dharmafect 2 (Dharmacon; catalogue no. T-2002-03) was used for SCC12 siRNA transfection. Plasmid transfections were performed using Fugene-6. Human Rab11b and 21 cDNAs were fused on to the C terminus of mCherry and CFP respectively (details available on request). GFP-Rab5a was a gift from Sharon Tooze (Cancer Research UK London Research Institute).

The following siRNA reagents from Dharmacon were used: RabGGTase*β* #1, GAGAAUGAGUGGCAUCUAUUU; RabGGTase*β* #2, UUACUUGGCUGGUGGCUUUUU; Rab5a #1, GCUAUGAACGUGAAUGAUCUU; Rab5a #2, CAAGCCUGGUAUUACGUUUUU; Rab5a #3, ACAAACGUAUGGUGGAGUAUU; Rab11b #1, GACAGAAGCCCAACAAGCUUU; Rab11b #2, GGAUUCCACUAACGUAGAGUU; Rab11b #3, UAACGUAGAGGAAGCAUUCUU; Rab21 #1, GGAGAGACAUGUUUCCAUUUU; Rab21 #2, GAGUAAACCUUGCCAUAUGUU; Rab21 #4, CAAGAGAGAUUCCAUGCAUUU; Cdc42 #1, GUGGGUGCCUGAGAUAACUUU; Cdc42 #2, AGACUCCUUUCUUGCUUGUUU; Integrin *α*5 #1, GAACGAGUCAGAAUUUCGAUU; Integrin *α*5 #2, CAAACGCUCCCUCCCAUAUUU; RabGGTase*β* smart pool #M-007262-01; Rab4a smart pool #M-008539–01; Rab4b smart pool #M-008780-03; Rab5a smart pool #M-004009-00; Rab5b smart pool #M-004010-01; Rab8a smart pool #M-003905-00; Rab8b smart pool #M-008744-01; Rab11a smart pool #M-004726-02; Rab11b smart pool #M-004727-02; Rab21 smart pool #M-009450-01.

### Compound screening

The ‘library’ of compounds for screening is described with Supplementary Table 1. A total of 2.5 × 10^3^ CAFs were embedded in 1 ml of a mixture of collagen I and Matrigel yielding a final collagen I concentration of approximately 4.6 mg ml^−1^ and a final Matrigel concentration of 2.2 mg ml^−1^. Gel mix (100 *μ*l) per well was plated in a 96-well plate. After the gel was set at 37°C for 1 h, 100 *μ*l media containing inhibitors or growth factors (see above) were added on top of the gel at either 1 or 10 *μ*M final concentration for 4 days with 100 *μ*l top up after 2 days. After 4 days, the plates were scanned and the diameter of the gel to well was measured using ImageJ software (http://rsbweb.nih.gov/ij/). The extent of contraction was assessed by subtracting the gel area from the well area. For each plate, the contraction values were normalised to the average contraction in the eight DMSO wells.

### Microscopy

Cells were either plated on glass coverslips or seeded into a gel consisting of 2–2.5 mg ml^−1^ (BD Biosciences; catalogue no. 354234) Matrigel and 4.25–4.75 mg ml^−1^ collagen I (BD Biosciences; catalogue no. 354249). Cells were fixed using 4% paraformaldehyde in PBS and then permeablised using 0.25% Triton X-100 in PBS before blocking with 3% bovine serum albumin in PBS. The following primary antibodies were used diluted 1 : 100: anti-pS19-MLC2 (Cell Signaling; catalogue no. 3671), anti-*α*5 integrin (Santa Cruz; catalogue no. sc-13547), anti-Rab5a (Abcam; catalogue no. ab18211). Fluorescent time-lapse imaging used MARS-lifeact (Riedl *et al*, 2008) to highlight F-actin and CFP-Rab21. All fluorescent images were acquired using Zeiss LSM 510 confocal microscopes (Jena, Germany).

### Organotypic invasion assays

Two types of organotypic assay were performed: either both cell types were alive simultaneously as described in Gaggioli *et al* (2007), or to more specifically evaluate the effect of compounds on either the CAFs or the carcinoma cells, the assay was divided into two phases. During the first phase CAFs were allowed 90–96 h to remodel the matrix in the absence of SCC12 cells before killing with 10 *μ*g ml^−1^ puromycin. During this phase the matrix can be remodelled by fibroblasts leading to its contraction: at the start of the puromycin treatment the matrix was photographed to enable quantification of the extent of contraction. After extensive washing of the ECM with media for 24 h SCC12, cells were plated on top of the matrix that was mounted on a bridge at a gas/liquid interface and left for 5 days. Inhibitors could be added during either phase. Also all siRNA experiments were performed using this two-phase experimental design.

### Western blotting

Western blots were performed using standard techniques. The following antibodies were used: Rab5a (Abcam; catalogue no. ab18211), Rab21 (Santa Cruz; catalogue no. sc-81917), RabGGTase*β* subunit (Abnova; catalogue no. h00005876-mo2), integrin *α*5 (Santa Cruz; catalogue no. sc-10729), pS19-MLC2 Cell Signaling (catalogue no. 3671) and MLC Cell Signaling (catalogue no. 3672).

### Adhesion assays

Adhesion assays were performed as described by Pinner and Sahai (2008).

### Flow cytometry

Analysis was performed as described by Brockbank *et al* (2005) using anti-*α*5 integrin (Santa Cruz; catalogue no. sc-13547) on non-permeablised cells.

## Results

### A chemical screen identifies statins as reducing matrix remodelling

**Figure 1:**
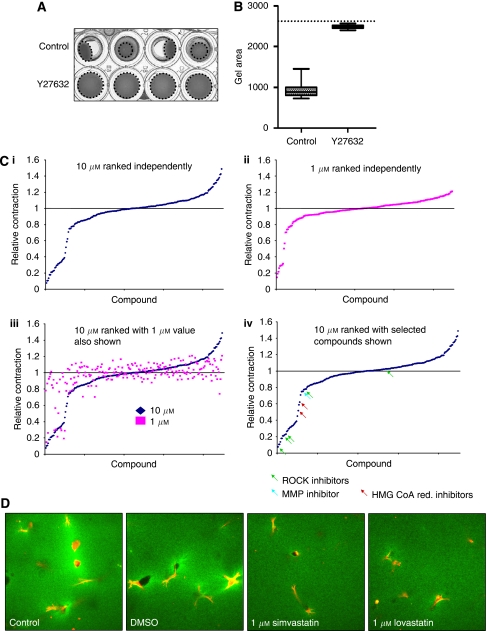
Chemical screen to identify compounds that block fibroblast-promoted matrix remodeling. (**A**) Replicates are shown of CAFs contracting ECM over a 4-day period in 96-well plates in the absence or presence of 10 *μ*M Y27632. (**B**) Quantification of the gel area of eight replicates of the gel contraction assay ±10 *μ*M Y27362. The dotted line represents the area of the well, which is equivalent to the gel area at the start of the assay. (**C**) (i) Range of normalised gel contraction values is shown following 10 *μ*M treatment of CAFs for 4 days. Median=1.002; mean=0.979 and standard deviation=0.080 for empty control wells. (ii) Range of normalised gel contraction values is shown following 1 *μ*M treatment of CAFs for 4 days. (iii) Range of normalised gel contraction values is shown along the *x* axis following 10 *μ*M treatment (blue) of CAFs for 4 days together with the extent of gel contraction following 1 *μ*M treatment with same compound (pink). (iv) Range of normalised gel contraction values is shown following 10 *μ*M treatment of CAFs for 4 days with selected classes of compounds highlighted. (**D**) Reflectance images of the extracellular matrix (green) and carcinoma-associated fibroblasts (F-actin staining in red) are shown. Cultures were treated with DMSO, 1 *μ*M simvastatin or 1 *μ*M lovastatin for 48 h.

Our previous work had shown a correlation between microscopic matrix remodelling and macroscopic gel contraction (Gaggioli *et al*, 2007). Further analysis showed a positive correlation between matrix contraction by CAFs and their ability to promote SCC invasion (Supplementary Figure 1). This led us to speculate that compounds that block matrix contraction would also block carcinoma cell invasion promoted by CAFs. To test this idea, we developed a high-throughput assay for matrix contraction. Carcinoma-associated fibroblasts consistently contract a collagen/Matrigel matrix in a 96-well plate over a 4-day period. Matrix contraction is blocked by a ROCK inhibitor, Y27632, which reduces actomyosin contraction (Figure 1A). This effect was simply quantified by measuring the area of the gel (Figure 1B).

We used 96-well plate assay to screen 182 inhibitors, growth factors and cytokines for their ability to modulate collagen contraction at both 1 and 10 *μ*M. The screen was performed twice and for each plate the matrix contraction was normalised to the average of the DMSO control points before the average of the two experiments was calculated. There were also 20 empty wells included in the screening plates and from these we estimated the normalised standard deviation of the assay as 0.08. Figure 1C, i and ii shows the range of normalised scores following both 1 and 10 *μ*M treatment. A smaller range of deviation from the controls was observed following 1 *μ*M treatment; although several compounds reduced collagen contraction at both concentrations (Figure 1C, iii). Compounds that reduced contraction to less than 0.6 at 1 *μ*M also reduced contraction at 10 *μ*M but that the converse was not necessarily true. The complete data set is listed in Supplementary Table 1; whereas selected classes of compounds are shown in Figure 1C, iv. Four out of the five compounds included in the screen reported to inhibit ROCK1 and 2 strongly reduced collagen contraction (including the well-established ROCK inhibitors Y27632 and H1152). The broad-spectrum MMP inhibitor GM6001 also inhibited matrix contraction. Interestingly, two HMG-CoA reductase inhibitors (simvastatin and lovastatin, generically referred to as statins) reduced collagen contraction by ∼0.4 and ∼0.1 normalised units at 10 and 1 *μ*M respectively (highlighted with red arrowheads in Figure 1C, iv). Statins are widely used to treat hypercholesterolaemia and have been associated with reduced cancer incidence and/or mortality (Demierre *et al*, 2005). We therefore decided to investigate their action on matrix remodelling by CAFs further.

**Figure 2:**
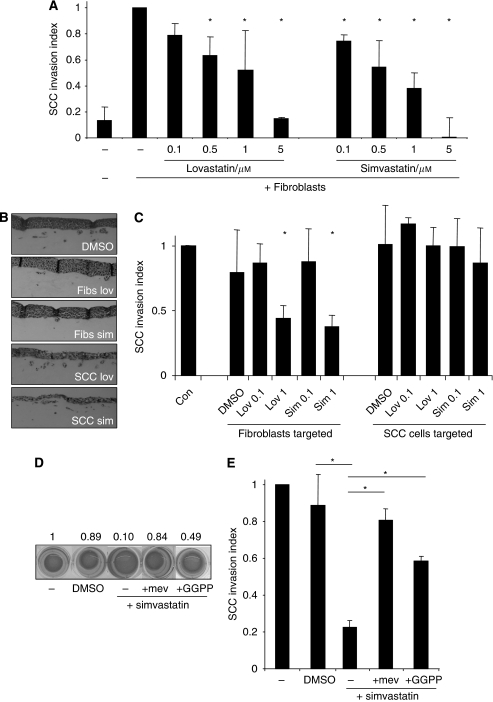
Statins prevent fibroblasts from promoting the invasion of carcinoma cells. (**A**) Invasion of organotypic cultures of SCC12 cells overlaid on top of a collagen-rich matrix containing CAFs (where indicated) were treated with the indicated dose of either simvastatin or lovastatin. The extent of SCC12 invasion is shown: values were normalised to the +CAF invasion index and the average of 10 fields from two experiments is shown. ^*^P<0.01, Student's *t*-test. (**B**) Representative images showing SCC12 invasion into a collagen-rich matrix with either fibroblasts treated with 1 *μ*M lovastatin or simvastatin during the CAF remodeling phase or SCC12 cells treated during the invasion phase of the assay. (**C**) CAFs were allowed to remodel a collagen-rich matrix before they were killed and SCC12 cells were overlaid on the remodelled matrix. The indicated dose of simvastatin or lovastatin (*μ*M) was added either during the CAF remodeling phase (left-hand side) or during the SCC12 invasion phase (right-hand side). The extent of SCC12 invasion is shown: values were normalised to the +CAF invasion index and the average of 10 fields from two experiments is shown. ^*^P<0.01, Student's *t*-test. (**D**) Matrix contraction by CAFs after 4 days is shown following the indicated treatments (1 *μ*M simvastatin, 20 *μ*M GGPP, 400 *μ*M mevalonate). Numbers represent the decrease in area of the matrix normalised to the control. (**E**) CAFs were allowed to remodel the matrix in the presence of the indicated treatments before they were killed and SCC12 cells were overlaid on the remodelled matrix. The extent of SCC12 invasion is shown: values were normalised to the +CAF invasion index and the average of 10 fields from two experiments is shown. ^*^P<0.01, Student's *t*-test.

We confirmed that the defects in collagen contraction observed macroscopically reflected defects in matrix re-organisation at the microscopic level. Control and DMSO-treated fibroblasts were able to both locally concentrate matrix (increased green signal around cells) and generate large holes (dark areas) in the matrix (Figure 1D). In contrast, these activities were defective in simvastatin- or lovastatin-treated fibroblasts. We next tested whether statins affect CAF-promoted SCC invasion. Figure 2A shows that both statins reduce SCC invasion in an organotypic model with IC_50_ values approximately 0.5–1 *μ*M. Supplementary Figure 2 shows that neither cell growth nor survival was significantly affected by statin doses below 5 *μ*M. The organotypic assay used to assess invasion depends on two cell types, the SCC cells and CAFs, either one of which could be affected by statin treatment. To determine which cell type is critically affected by statins, we used a modified assay in which fibroblast-mediated matrix remodelling and SCC invasion into the matrix occur sequentially (Gaggioli *et al*, 2007). Statins were then applied either during the fibroblast-promoted matrix remodelling phase or the SCC invasion phase when carcinoma cells move into a matrix that has previously been ‘conditioned’ by CAFs. Figure 2B and C shows that 1 *μ*M simvastatin or lovastatin treatment during the matrix remodelling phase reduces SCC invasion but treatment after this phase has no effect. These data indicate that statins prevent CAFs from generating a matrix that is permissive for SCC invasion and not by preventing SCC cells invading an already permissive matrix.

### Rab protein lipid modification is required for matrix remodelling

**Figure 3:**
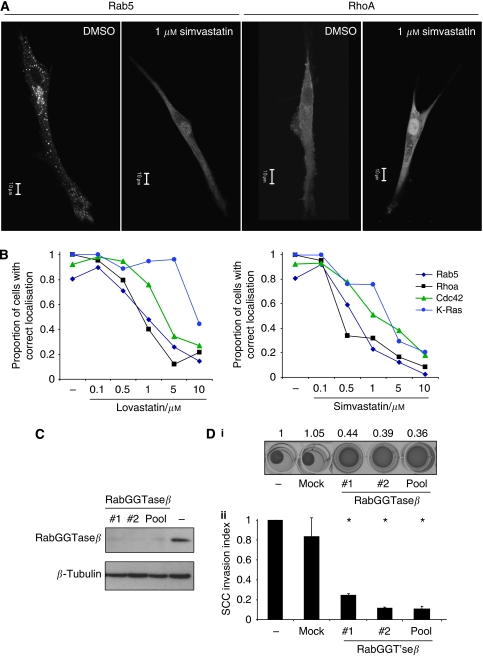
Rab protein modification is a key target of statin action. (**A**) Images of GFP-Rab5 and GFP-RhoA localisation in CAFs are shown in the absence or presence of 1 *μ*M simvastatin. (**B**) Quantification of the proportion of CAFs with correct subcellular targeting of mRFP-Cdc42 (plasma membrane), GFP-RhoA (plasma membrane), GFP-Rab5a (endosomal) and mRFP-K-ras C-term (plasma membrane) at the indicated doses of lovastatin or simvastatin. (**C**) Western blot showing levels of RabGGTase*β* subunit following transfection of smart-pool siRNAs # 1 and # 2 targeting RabGGTase*β* subunit. *β*-Tubulin is shown as a loading control. (**D**) (i) Matrix contraction by CAFs after 4 days is shown following transfection of smart-pool siRNAs #1 and #2 targeting RabGGTase*β* subunit. (ii) CAFs were allowed to remodel a collagen-rich matrix following transfection of smart-pool siRNAs #1 and #2 targeting RabGGTase*β* subunit before they were killed and SCC12 cells were overlaid on the remodelled matrix. The extent of SCC12 invasion is shown: values were normalised to the +CAF invasion index and the average of 10 fields from two experiments is shown. ^*^P<0.01, Student's *t*-test.

Statins modulate cholesterol biosynthesis indirectly and also affect the production of many other metabolites (Werner *et al*, 2002; Walker and Olson, 2005). In particular, the biosynthesis of mevalonate, geranyl-, farnesyl- and geranylgeranyl-pyrophosphate is affected by statin treatment. To investigate the mechanism of statin action in more detail, we attempted to ‘rescue’ the effect of statin treatment by adding additional mevalonate or geranylgeranyl-pyrophosphate. As expected, mevalonate restored both matrix contraction by CAFs and their ability to generate matrix permissive for SCC12 invasion (Figure 2D and E). In addition, geranylgeranyl-pyrophosphate significantly restored these activities in CAFs (Figure 2D and E). This finding is striking because geranylgeranyl-pyrophosphate is not an intermediate between HMG-CoA reductase and cholesterol synthesis but is largely used to modify small G proteins and enable their interaction with cellular membranes. Furthermore, previous reports have documented defects in small G-protein targeting after statin treatment (Laezza *et al*, 1998; Kusama *et al*, 2001; Holstein *et al*, 2002; Ghittoni *et al*, 2005; Walker and Olson, 2005). In agreement with these findings, simvastatin and lovastatin both affected the subcellular localisation of a range of small G proteins in CAFs (Figure 3A and B). Of the proteins investigated, the subcellular targeting of Rab5 and RhoA was most sensitive to statin treatment with pronounced effects observed at doses that also reduce fibroblast-stimulated SCC invasion (1 *μ*M). We have previously shown a role for RhoA in matrix remodelling by CAFs (Gaggioli *et al*, 2007) but were intrigued by the possibility that Rab proteins may also be critical for this process.

**Figure 4:**
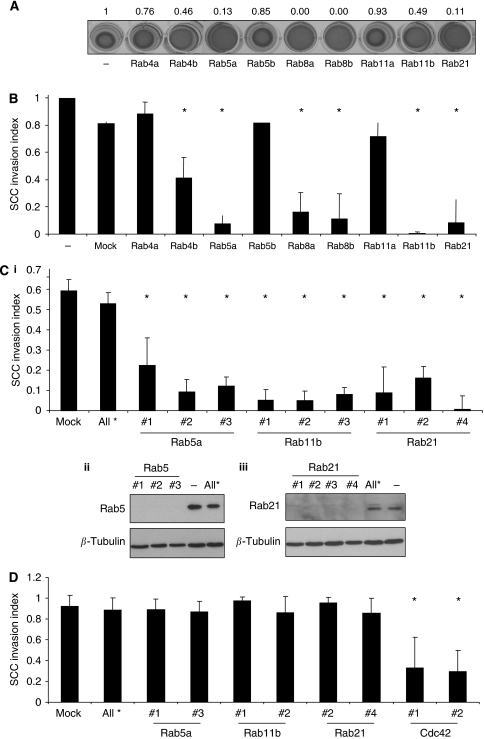
Rabs 5a, 11b and 21 are required for fibroblasts to promote SCC invasion. (**A**) Matrix contraction by CAFs after 4 days is shown following transfection of smart-pool siRNAs against the Rabs indicated. (**B**) CAFs were allowed to remodel a collagen-rich matrix following transfection of smart-pool siRNAs against the Rabs indicated before they were killed and SCC12 cells were overlaid on the remodelled matrix. The extent of SCC12 invasion is shown: values were normalised to the +CAF invasion index and the average of 10 fields from two experiments is shown, error bars represent half range of the data. ^*^P<0.01, Student's *t*-test. (**C**) (i) CAFs were allowed to remodel the matrix following transfection of single siRNAs against the Rabs indicated before they were killed and SCC12 cells were overlaid on the remodelled matrix. The extent of SCC12 invasion is shown: values were normalised to the +CAF invasion index and the average of 10 fields from two experiments is shown, error bars represent half range of the data. ^*^P<0.01, Student's *t*-test. (ii) Western blot showing levels of Rab5a following transfection of siRNAs #1, #2 and #3 targeting Rab5a. *β*-Tubulin is shown as a loading control. (iii) Western blot showing levels of Rab21 following transfection of siRNAs #1, #2 #3 and #4 targeting Rab21. *β*-Tubulin is shown as a loading control. (**D**) Invasion of SCC12 cells following transfection of single siRNAs against the Rabs indicated (or Cdc42) into a CAF-conditioned matrix is shown. Invasion index is relative to untreated controls. Average of 10 fields from two experiments is shown, error bars represent half range of the data. ^*^P<0.01, Student's *t*-test.

**Figure 5:**
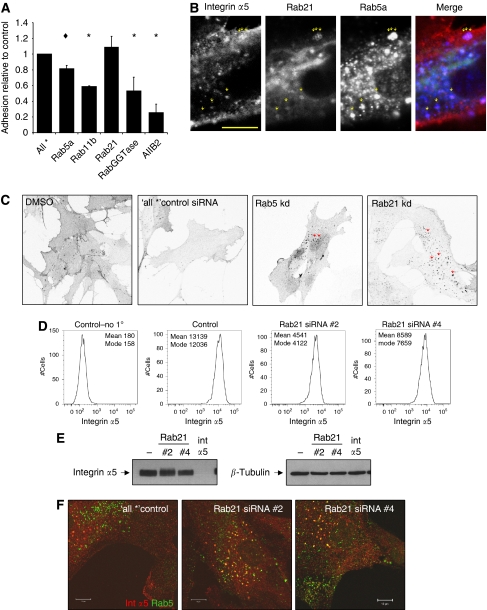
Rab21 is required for integrin *α*5 to exit Rab5-positive vesicles. (**A**) CAF adhesion to a mixture of collagen I/Matrigel is shown following transfection of smart-pool siRNAs against the Rabs indicated or incubation with integrin *β*1 blocking antibody, AIIB2. Average of six fields from two experiments is shown, error bars represent half range of the data. ^*^P<0.01, Student's *t*-test; ♦*P*<0.05, Student's *t*-test. (**B**) Localisation of CFP-Rab21 (green in merge), endogenous Rab5a (blue in merge) and endogenous integrin *α*5 (red in merge) in a CAF cultured within a three-dimensional matrix of collagen I/Matrigel. Scale bar=10 *μ*m. (**C**) Localisation of integrin *α*5 in CAFs following transfection of smart-pool siRNAs against the Rabs indicated. Red arrowheads indicate intracellular vesicles. (**D**) Flow cytometry analysis of integrin *α*5 levels on the surface of CAFs following transfection of siRNA targeting Rab21. (**E**) Western blots showing levels of integrin *α*5 and *β*-tubulin following transfection of CAFs with Rab21 or integrin *α*5 siRNA. (**F**) Localisation of integrin *α*5 (red) and Rab5a (green) in CAFs following transfection of single siRNAs against Rab21 or ‘all ^*^’ control.

To test whether the geranylgeranyl modification of Rab proteins is required for CAFs to promote SCC invasion, we depleted the enzyme that adds the lipid groups to Rab proteins. Two independent siRNA to the RabGGTase*β* subunit effectively depleted that protein and also prevented fibroblasts from contracting the ECM (Figure 3C and D). Furthermore, siRNA against RabGGTase*β* subunit dramatically reduced the ability of CAFs to promote the invasion of SCC cells (Figure 3D). These data show that lipid modification of Rab proteins is critical for CAF-promoted cancer cell invasion. We next wished to determine which Rabs might be required for CAFs to promote SCC invasion. We therefore screened numerous Rabs that have been implicated in cytoskeletal regulation, integrin or MMP trafficking. Figure 5A shows that siRNA smart pools to Rab4b, Rab5a, Rab8a, Rab8b, Rab11b and Rab21 reduced the ability of CAFs to contract the ECM (Figure 4A). Correspondingly, all these Rabs except Rab4a reduced CAF-promoted SCC invasion (Figure 4B). The results for Rab5a, Rab11b and Rab21 could be reproduced using at least three independent siRNA targeting sequences (Figure 4C, i). The efficiency of siRNA knockdown of Rab5a and Rab21 is shown in Figure 4C, ii and iii. Interestingly, depletion of Rab5a, 11b or 21 in carcinoma cells did not affect their ability to invade into matrix that had previously been remodelled by fibroblasts (Figure 4D).

### Rab21 is required for integrin *α*5 accumulation at the plasma membrane

Rab5a, 11 and 21 have all been implicated in the recycling of integrins, although Rab11 is implicated in a different recycling route from Rabs5a and 21 (Jones *et al*, 2006; Pellinen *et al*, 2006). In agreement with this we found that there was extensive colocalisation between Rab5a and Rab21 but not with Rab11b (Supplementary Figure 3). To learn more about the role of these Rabs, we performed time-lapse imaging of control and Rab-depleted CAFs plated on deformable collagen-rich matrices. Mock- or control-transfected CAFs exhibited broad cell protrusions that were either associated with cell motility or local deformation of the matrix (Supplementary Movie 1). In contrast, Rab5a-, 11b- or 21-depleted cells had more transient protrusions that were not usually associated with matrix deformation or movement (Rab21siRNA shown in Supplementary Movie 2). To determine if depletion of Rab5a, Rab11b or Rab21 was simply preventing fibroblasts from adhering to the matrix, we performed cell adhesion assays. Adhesion to the collagen I/Matrigel mixture used in our assays was reduced to varying degrees by depletion of Rab GGTase*β*, Rab11b and Rab5a, and by an integrin *β*1 blocking antibody (Figure 5A). Interestingly, siRNA against Rab21 had no effect: therefore, defects in matrix remodelling in the absence of Rab21 are not due to a failure to adhere to the ECM (Figure 5A). This led us to focus our attention on Rab21. We confirmed that low doses of statins disrupted the subcellular localisation of Rab21 (Supplementary Figure 4).

We next investigated if Rab21 was having a more specific effect on the cell adhesion machinery. Analysis of the location of a range of integrins revealed that a fraction of integrin *α*5 colocalised with Rab5a and Rab21 in fibroblasts in three-dimensional matrices (Figure 5B). In many cases Rab21 appeared to be most concentrated around the outside of the Rab5a- and integrin *α*5-positive vesicles (in top part of panels, marked with arrowheads). We therefore investigated if Rab5a or Rab21 were involved regulating integrin *α*5 in CAFs. Depletion of Rab21 and, to a lesser extent, Rab5a led to accumulation of integrin *α*5 in internal vesicular structures with slightly lower levels of plasma membrane staining (Figure 5C, marked with red arrowheads). We used quantitative flow cytometry to confirm the reduction in integrin *α*5 levels at the plasma membrane following Rab21 depletion (Figure 5D). In the absence of Rab21 integrin *α*5 accumulates in intracellular vesicles positive for Rab5 (Figure 5E) and EE1A (data not shown). Total integrin *α*5 levels were not significantly affected by Rab21 siRNA (Figure 5E). We did not see any dramatic changes in the distribution of integrin *β*1, *α*3 or *α*V in Rab21-depleted cells (Supplementary Figure 5), nor did we observe affects on cell proliferation following Rab21 knockdown (Supplementary Figure 6A). Together these data suggest that Rab21 is required for efficient delivery of integrin *α*5 to the plasma membrane.

**Figure 6:**
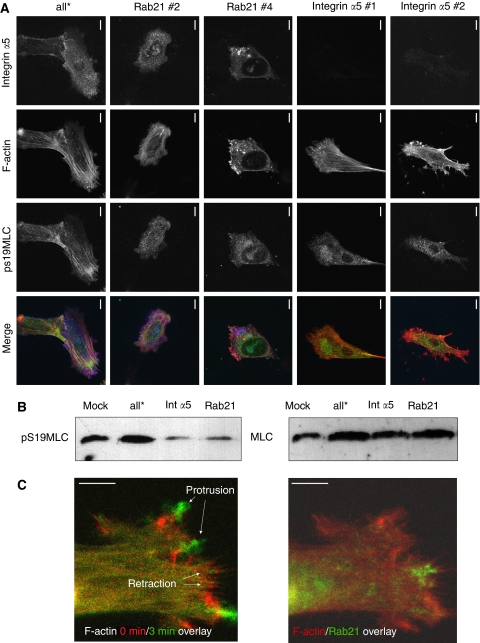
Rab21 and integrin *α*5 are required for actomyosin cables. (**A**) Panels show the organisation of integrin *α*5 (blue in merge), F-actin (red in merge) and pS19-MLC (green in merge) in CAFs plated on a mixture of collagen I/Matrigel. Scale bar=20 *μ*m. (**B**) Western blots show pS19-MLC and total MLC levels in CAFs plated on collagen-rich gels following transfection of siRNA targeting Rab21 or integrin *α*5. (**C**) Left-hand panel shows the actin organisation in a CAF plated on a mixture of collagen I/Matrigel at two time points 3 min apart (*t*=0 is in red and *t*=3 is in green). Areas of protrusion and retraction are indicated. Right-hand panel shows the distribution of actin (red) and Rab21 (green) in the same cell at *t*=0. Scale bar=10 *μ*m.

ROCK-dependent regulation of actomyosin contractility is critical for matrix remodelling (Figure 1; Gaggioli *et al*, 2007). We therefore tested whether fibroblasts lacking Rab21 or integrin *α*5 had defects in the activation of the actomyosin contractile machinery. Control cells typically had an elongated morphology with thick actin cables associated with phosphorylated ‘active’ MLC running along their length. Strikingly, cells depleted for Rab21 had a less elongated morphology (quantification is shown in Supplementary Figure 6B). Rab21-depleted cells also lacked contractile actomyosin cables spanning the cell body, although phosphorylated MLC could still be detected (Figure 6A). Fibroblasts depleted for integrin *α*5 also had reduced levels of phosphorylated MLC associated with F-actin cables. Western blot analysis confirmed that Rab21 and integrin *α*5 regulate MLC phosphorylation in CAFs (Figure 6B). We further probed the relationship between Rab21 and cell contractility by analysing Rab21 dynamics in CAFs plated on deformable matrices. Figure 6C shows that Rab21 is much more prominently localised in areas of the cell that are contracting compared to those that are protruding (also Supplementary Movie 3). These data suggest that the localised delivery of Rab21 cargoes, such as integrin *α*5, can promote the generation of contractile force.

**Figure 7:**
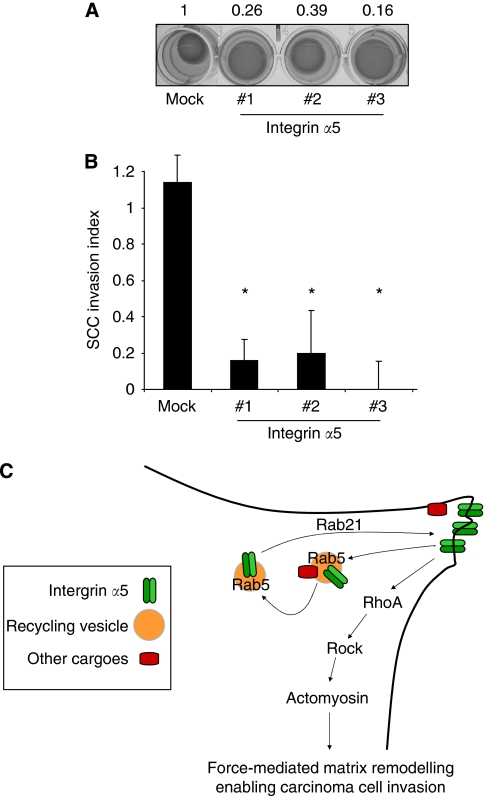
Integrin *α*5 is required for CAFs to contract gels and promote SCC invasion. (**A**) Matrix contraction by CAFs after 4 days is shown following transfection of smart-pool siRNAs against integrin *α*5. (**B**) CAFs were allowed to remodel a collagen-rich matrix following transfection of siRNA against integrin *α*5. The extent of SCC12 invasion is shown: values were normalised to the +CAF invasion index and the average of 10 fields from two experiments is shown, error bars represent half range of the data. ^*^P<0.01, Student's *t*-test. (**C**) Rab21 functions locally to aid the delivery of integrin *α*5 to the plasma membrane. Integrin *α*5 then promotes actomyosin contractility that can be used to remodel the surrounding matrix.

To determine whether reduced integrin *α*5 function could account for the reduced ability of Rab21-depleted CAFs to contract the matrix and promote SCC invasion, we investigated the effect of integrin *α*5 depletion. Figure 7A shows that depletion of integrin *α*5 reduces matrix contraction by CAFs (efficacy of siRNAs #1 and 2 shown in Figure 6A). Correspondingly, integrin *α*5 depletion also reduces the ability of CAFs to promote SCC invasion (Figure 7B). These data support integrin *α*5 delivery to the plasma membrane as a key function of Rab21 in enabling CAF-promoted SCC invasion.

## Discussion

Matrix remodelling by fibroblasts is critical for normal and pathological processes including wound healing, fibrosis and cancer invasion (Grinnell, 2003). One manifestation of matrix remodelling is contraction of collagen gels (Ngo *et al*, 2006). We noted a correlation between CAF-promoted matrix contraction and their ability to promote the invasion of carcinoma cells. Therefore, we screened ∼200 compounds to identify agents that would prevent matrix contraction with the hope that they would also reduce fibroblast-promoted carcinoma invasion. Numerous compounds were identified in this screen; however, many were discarded when we tested their effect on cell viability. We also noticed that many compounds that reportedly have the same molecular target gave different matrix contraction scores (Supplementary Table 1). This may reflect differing stabilities of the compounds in the 4-day matrix contraction assay, varying degrees of efficacy or off-target effects.

We focused on two related agents that scored similarly in the matrix contraction assay, simvastatin and lovastatin. As we hypothesised, these compounds also reduced the ability of CAFs to promote cancer cell invasion. Statins are of interest because several studies have shown reduced cancer incidence or mortality in patients taking these drugs for elevated cholesterol (Demierre *et al*, 2005). Several mechanisms have been put forward to account for these observations (Murtola *et al*, 2008). Here, we show that statins reduce cancer invasion by targeting the stromal compartment. Specifically, statins reduce the ability of CAFs to remodel the matrix in a way that enables carcinoma cell invasion. Importantly, this effect occurs at doses (0.5 and 1 *μ*M) that are not cytotoxic or cytostatic and are close to those observed in cancer patients (Holstein *et al*, 2006). Consistent with this, statins can reduce the activation of other types of fibroblast and matrix contraction (Meyer-Ter-Vehn *et al*, 2008; Burke *et al*, 2009), although Rab proteins were not implicated. This suggests reduced matrix remodelling by the stroma could partly account for the altered cancer incidence in people taking statins. However, this study does not exclude other mechanisms.

Further analysis led us to conclude that cholesterol is not the critical target involved in matrix remodelling but to focus on proteins that have GGPP modifications. At least three Rab proteins (5a, 11b and 21) are required in CAFs for their ability to promote SCC invasion. Depletion of these Rabs did not affect the ability of SCC cells to invade matrix that had previously been remodelled by CAFs; this is probably because the ‘following’ SCC cells do not remodel the ECM and therefore matrix remodelling pathways are dispensable in SCC cells. Subsequent experiments showed that Rab21 is specifically required for effective integrin *α*5 accumulation at the plasma membrane. When Rab21 is absent, integrin *α*5 accumulates in Rab5a-positive vesicles suggesting that Rab21 is required for exit from this compartment (Figure 7C). Provocatively, Rab21-positive vesicles are preferentially localised to areas of cell contraction and both Rab21 and integrin *α*5 are needed for MLC phosphorylation. These data led us to propose that Rab21 delivers integrin *α*5 to the plasma membrane where it locally signals to the contractile machinery. Interestingly, a connection between Rab21, integrins and actomyosin regulation has been reported during cytokinesis (Pellinen *et al*, 2006, 2008). This is probably through the activation of Rho–ROCK function as ligand-bound integrin *α*5 can signal to RhoGEFs and thereby increase Rho activity (Ren *et al*, 1999; Dubash *et al*, 2007; Huveneers *et al*, 2008). Integrin *α*5 has also been implicated in collagen gel contraction in other fibroblast types (Liu *et al*, 2006). In these experiments it appears that other integrins are unable to compensate for loss of integrin *α*5 function. Our data do not exclude the possibility that Rab21 is required for the delivery or trafficking of other critical targets, in addition to integrin *α*5.

The analysis performed here illustrates that targeting of the stroma alone can reduce the invasion of cancer cells. Unfortunately, it also implies that the implementation of anti-invasion agents that target matrix remodelling may only be of limited use if the matrix surrounding a tumour is already permissive for carcinoma invasion. Nonetheless, statins may be useful in slowing the progression of early stage lesions and in range of other pathologies associated with aberrant fibroblast-mediated matrix remodelling, such as scarring and fibrosis. In conclusion, we have shown the feasibility of intermediate- to high-throughput screening of compounds that block matrix remodelling by fibroblasts. Following on from this, we have shown that statins reduce SCC invasion by targeting matrix remodelling by CAFs and have uncovered a critical role for Rab21 in this process.

## References

[bib1] Brockbank EC, Bridges J, Marshall CJ, Sahai E (2005) Integrin beta1 is required for the invasive behaviour but not proliferation of squamous cell carcinoma cells *in vivo*. Br J Cancer 92: 102–112 15597106 10.1038/sj.bjc.6602255 PMC2361733

[bib2] Burke JP, Watson RW, Murphy M, Docherty NG, Coffey JC, O’Connell PR (2009) Simvastatin impairs smad-3 phosphorylation and modulates transforming growth factor beta1-mediated activation of intestinal fibroblasts. Br J Surg 96: 541–551 19358180 10.1002/bjs.6577

[bib3] Demierre MF, Higgins PD, Gruber SB, Hawk E, Lippman SM (2005) Statins and cancer prevention. Nat Rev Cancer 5: 930–942 16341084 10.1038/nrc1751

[bib4] Dubash AD, Wennerberg K, Garcia-Mata R, Menold MM, Arthur WT, Burridge K (2007) A novel role for Lsc/p115 RhoGEF and LARG in regulating RhoA activity downstream of adhesion to fibronectin. J Cell Sci 120: 3989–3998 17971419 10.1242/jcs.003806

[bib5] Egeblad M, Littlepage LE, Werb Z (2005) The fibroblastic coconspirator in cancer progression. Cold Spring Harb Symp Quant Biol 70: 383–388 16869775 10.1101/sqb.2005.70.007 PMC2580828

[bib6] Friedl P, Wolf K (2008) Tube travel: the role of proteases in individual and collective cancer cell invasion. Cancer Res 68: 7247–7249 18794108 10.1158/0008-5472.CAN-08-0784

[bib7] Gaggioli C, Hooper S, Hidalgo-Carcedo C, Grosse R, Marshall JF, Harrington K, Sahai E (2007) Fibroblast-led collective invasion of carcinoma cells with differing roles for RhoGTPases in leading and following cells. Nat Cell Biol 9: 1392–1400 18037882 10.1038/ncb1658

[bib8] Ghittoni R, Patrussi L, Pirozzi K, Pellegrini M, Lazzerini PE, Capecchi PL, Pasini FL, Baldari CT (2005) Simvastatin inhibits T-cell activation by selectively impairing the function of Ras superfamily GTPases. FASEB J 19: 605–607 15677697 10.1096/fj.04-2702fje

[bib9] Grinnell F (2003) Fibroblast biology in three-dimensional collagen matrices. Trends Cell Biol 13: 264–269 12742170 10.1016/s0962-8924(03)00057-6

[bib10] Holstein SA, Knapp HR, Clamon GH, Murry DJ, Hohl RJ (2006) Pharmacodynamic effects of high dose lovastatin in subjects with advanced malignancies. Cancer Chemother Pharmacol 57: 155–164 16133537 10.1007/s00280-005-0013-8

[bib11] Holstein SA, Wohlford-Lenane CL, Hohl RJ (2002) Consequences of mevalonate depletion. Differential transcriptional, translational, and post-translational up-regulation of Ras, Rap1a, RhoA, and RhoB. J Biol Chem 277: 10678–10682 11788600 10.1074/jbc.M111369200

[bib12] Honjo M, Tanihara H, Kameda T, Kawaji T, Yoshimura N, Araie M (2007) Potential role of Rho-associated protein kinase inhibitor Y-27632 in glaucoma filtration surgery. Invest Ophthalmol Vis Sci 48: 5549–5557 18055804 10.1167/iovs.07-0878

[bib13] Huveneers S, Truong H, Fassler R, Sonnenberg A, Danen EH (2008) Binding of soluble fibronectin to integrin alpha5 beta1-link to focal adhesion redistribution and contractile shape. J Cell Sci 121: 2452–2462 18611961 10.1242/jcs.033001

[bib14] Jones MC, Caswell PT, Norman JC (2006) Endocytic recycling pathways: emerging regulators of cell migration. Curr Opin Cell Biol 18: 549–557 16904305 10.1016/j.ceb.2006.08.003

[bib15] Kusama T, Mukai M, Iwasaki T, Tatsuta M, Matsumoto Y, Akedo H, Nakamura H (2001) Inhibition of epidermal growth factor-induced RhoA translocation and invasion of human pancreatic cancer cells by 3-hydroxy-3-methylglutaryl-coenzyme a reductase inhibitors. Cancer Res 61: 4885–4891 11406567

[bib16] Laezza C, Bucci C, Santillo M, Bruni CB, Bifulco M (1998) Control of Rab5 and Rab7 expression by the isoprenoid pathway. Biochem Biophys Res Commun 248: 469–472 9703948 10.1006/bbrc.1998.9007

[bib17] Liu Y, Yanai R, Lu Y, Kimura K, Nishida T (2006) Promotion by fibronectin of collagen gel contraction mediated by human corneal fibroblasts. Exp Eye Res 83: 1196–1204 16914141 10.1016/j.exer.2006.06.008

[bib18] Meshel AS, Wei Q, Adelstein RS, Sheetz MP (2005) Basic mechanism of three-dimensional collagen fibre transport by fibroblasts. Nat Cell Biol 7: 157–164 15654332 10.1038/ncb1216

[bib19] Meyer-Ter-Vehn T, Katzenberger B, Han H, Grehn F, Schlunck G (2008) Lovastatin inhibits TGF-beta-induced myofibroblast transdifferentiation in human tenon fibroblasts. Invest Ophthalmol Vis Sci 49: 3955–3960 18421080 10.1167/iovs.07-1610

[bib20] Murtola TJ, Visakorpi T, Lahtela J, Syvala H, Tammela T (2008) Statins and prostate cancer prevention: where we are now, and future directions. Nat Clin Pract Urol 5: 376–387 18542103 10.1038/ncpuro1146

[bib21] Ngo P, Ramalingam P, Phillips JA, Furuta GT (2006) Collagen gel contraction assay. Methods Mol Biol (Clifton, NJ) 341: 103–109 10.1385/1-59745-113-4:103 16799192

[bib22] Olson MF, Sahai E (2009) The actin cytoskeleton in cancer cell motility. Clin Exp Metastasis 26: 273–287, e-pub 23 May 2008 18498004 10.1007/s10585-008-9174-2

[bib23] Pellinen T, Arjonen A, Vuoriluoto K, Kallio K, Fransen JA, Ivaska J (2006) Small GTPase Rab21 regulates cell adhesion and controls endosomal traffic of beta1-integrins. J Cell Biol 173: 767–780 16754960 10.1083/jcb.200509019 PMC2063892

[bib24] Pellinen T, Ivaska J (2006) Integrin traffic. J Cell Sci 119: 3723–3731 16959902 10.1242/jcs.03216

[bib25] Pellinen T, Tuomi S, Arjonen A, Wolf M, Edgren H, Meyer H, Grosse R, Kitzing T, Rantala JK, Kallioniemi O, Fassler R, Kallio M, Ivaska J (2008) Integrin trafficking regulated by Rab21 is necessary for cytokinesis. Dev Cell 15: 371–385 18804435 10.1016/j.devcel.2008.08.001

[bib26] Pinner S, Sahai E (2008) PDK1 regulates cancer cell motility by antagonising inhibition of ROCK1 by RhoE. Nat Cell Biol 10: 127–137 18204440 10.1038/ncb1675

[bib27] Ren XD, Kiosses WB, Schwartz MA (1999) Regulation of the small GTP-binding protein Rho by cell adhesion and the cytoskeleton. EMBO J 18: 578–585 9927417 10.1093/emboj/18.3.578 PMC1171150

[bib28] Riedl J, Crevenna AH, Kessenbrock K, Yu JH, Neukirchen D, Bista M, Bradke F, Jenne D, Holak TA, Werb Z, Sixt M, Wedlich-Soldner R (2008) Lifeact: a versatile marker to visualize F-actin. Nat Methods 5: 605–607 18536722 10.1038/nmeth.1220 PMC2814344

[bib29] Walker K, Olson MF (2005) Targeting Ras and Rho GTPases as opportunities for cancer therapeutics. Curr Opin Genet Dev 15: 62–68 15661535 10.1016/j.gde.2004.11.001

[bib30] Werner N, Nickenig G, Laufs U (2002) Pleiotropic effects of HMG-CoA reductase inhibitors. Basic Res Cardiol 97: 105–116 12002257 10.1007/s003950200000

[bib31] Yuge A, Nasu K, Matsumoto H, Nishida M, Narahara H (2007) Collagen gel contractility is enhanced in human endometriotic stromal cells: a possible mechanism underlying the pathogenesis of endometriosis-associated fibrosis. Hum Reprod 22: 938–944 17204524 10.1093/humrep/del485

