# Supplementary material for: A chemical biology screen reveals a role for Rab21-mediated control of actomyosin contractility in fibroblast-driven cancer invasion
Source: Br J Cancer. 2009 Dec 1;102(2):392–402. doi: 10.1038/sj.bjc.6605469 (PMC2816649; doi:10.1038/sj.bjc.6605469)
Supplement: Supplementary Figures 1–6 [file 6605469x1.pdf]

Supplementary Figure 1 Hooper et al.,

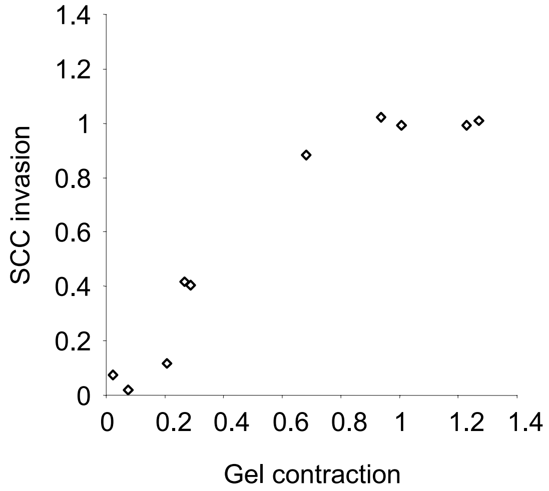

Supplementary Figure 1: Correlation between macroscopic matrix contraction by carcinoma associated fibroblasts and their ability to promote SCC invasion. Each point corresponds to a different siRNA treatment or inhibitor treatment (data for siRNA against integrins α1, α2, α3, α4, α5, αV and the following inhibitors 10μM Y27632, 10μM H1152, Tat-C3). Values for both gel contraction and SCC invasion are normalized to 1. Original data from Gaggioli et al., (1).

Supplementary Figure 2 Hooper et al.,

A

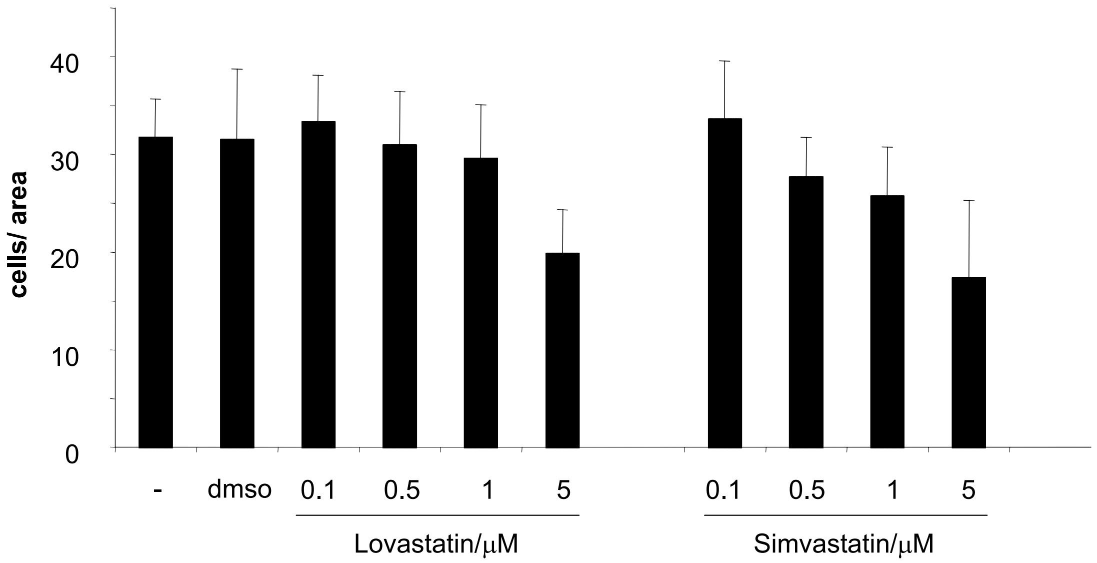

B

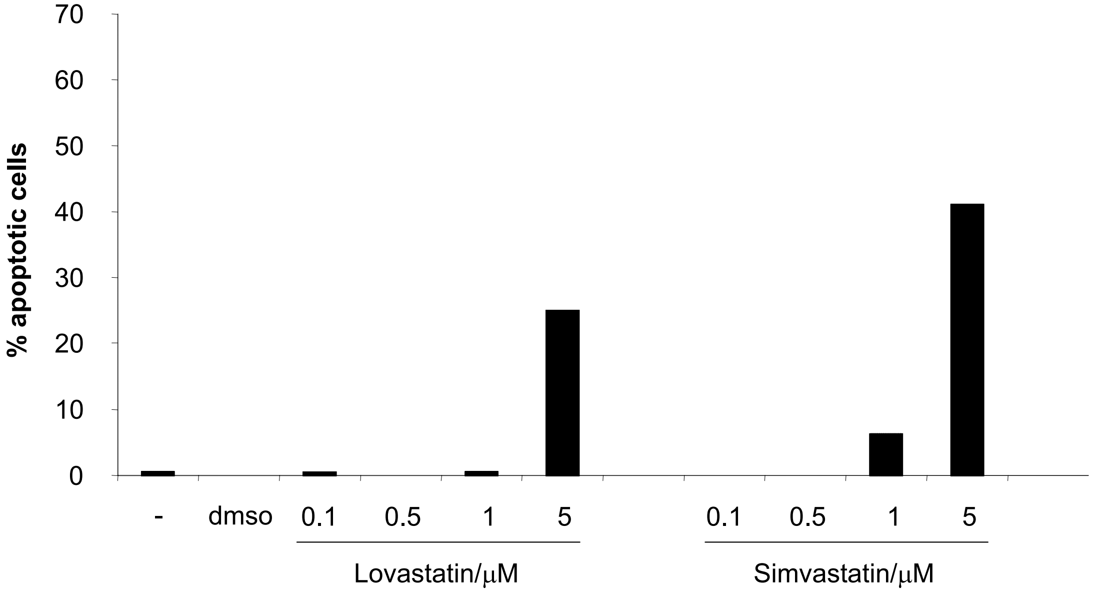

Supplementary Figure 2: A) Fibroblasts were plated on top of collagen I/matrigel matrices and treated with the indicated dose of statin for 4 days. The number of cells present per 20x microscope field was then determined - the average of three fields is shown. B) Fibroblasts were plated on top of collagen I/matrigel matrices and treated with the indicated dose of statin for 4 days. The number of apoptotic cells present per 20x microscope field was then determined based on nuclear morphology following DAPI staining - the average of three fields is shown.

Supplementary Figure 3 Hooper et al.,

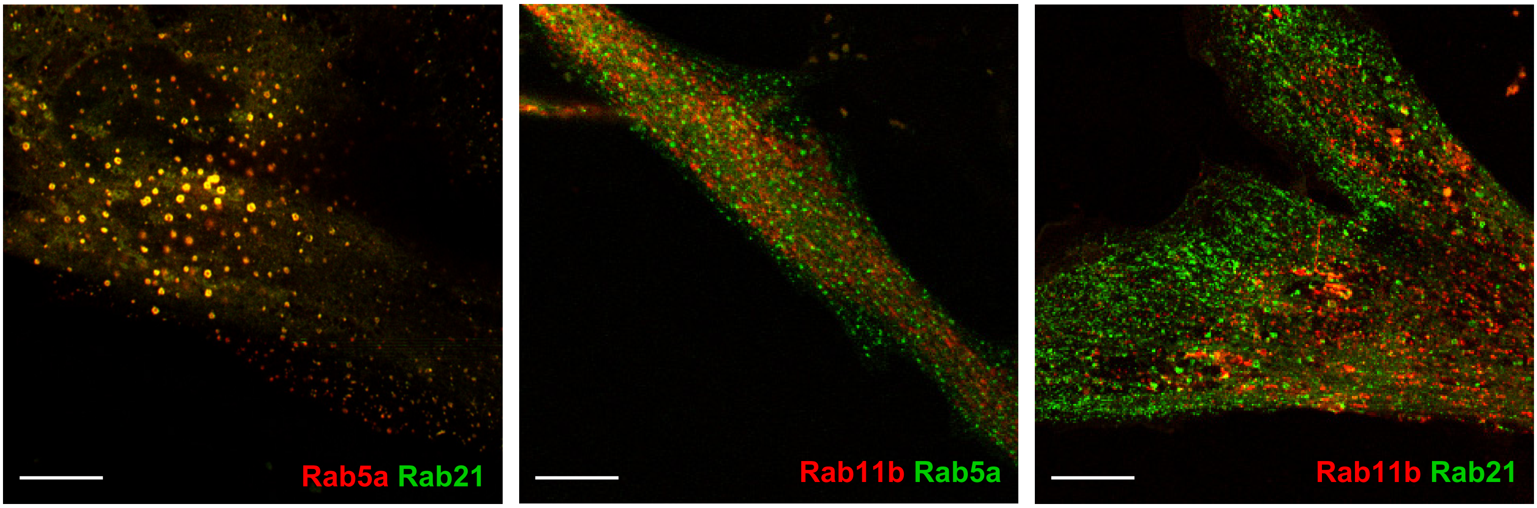

Supplementary Figure 3: The relative localisation of Rab5a, 11b and 21 in CAF’s is shown: left panel shows Rab5a (red) and Rab21 (green), middle panel shows Rab11b (red) and Rab21 (green) and right panel shows Rab11b (red) and Rab5a (green). Scale bar is 10μm.

Supplementary Figure 4 Hooper et al.,

A

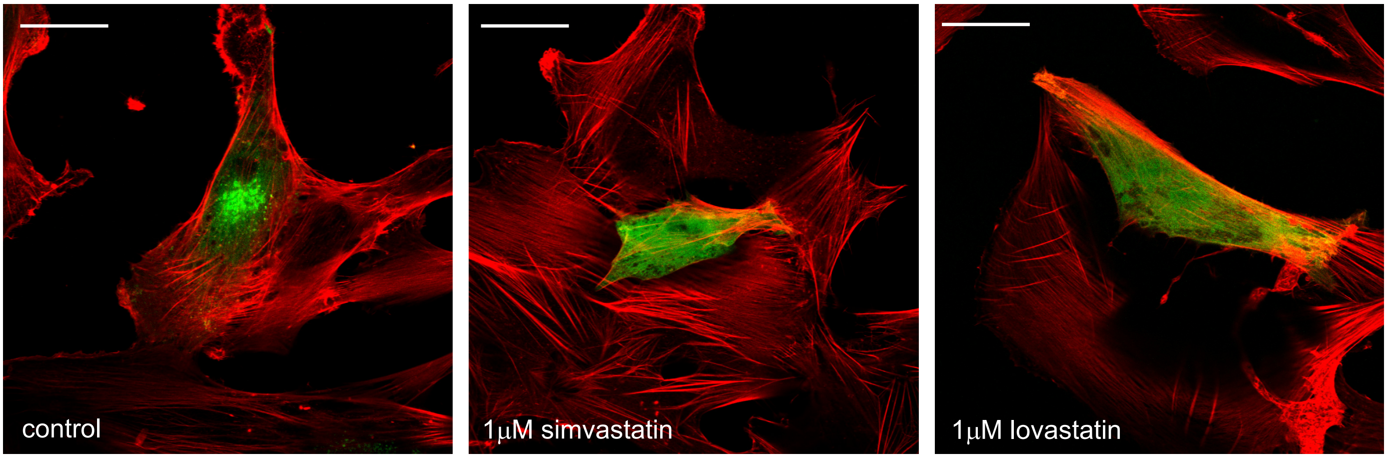

B

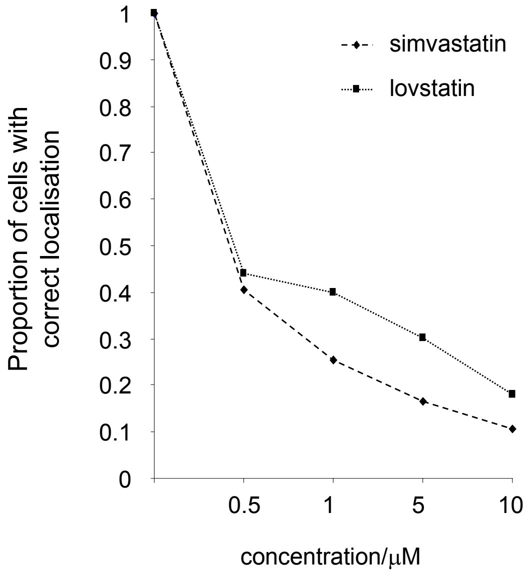

Supplementary Figure 4: A) The localisation of CFP-Rab21 is shown (in green) in control or 1μM simvastatin- or 1μM lovastatin-treated fibroblasts. F-actin is shown in red – scale bar is 40μm. B) Quantification of the proportion of CAF's with correct sub-cellular targeting of CFP-Rab21 at the indicated doses of lovastatin or simvastatin. Average of two experiments.

Supplementary Figure 5 Hooper et al.,

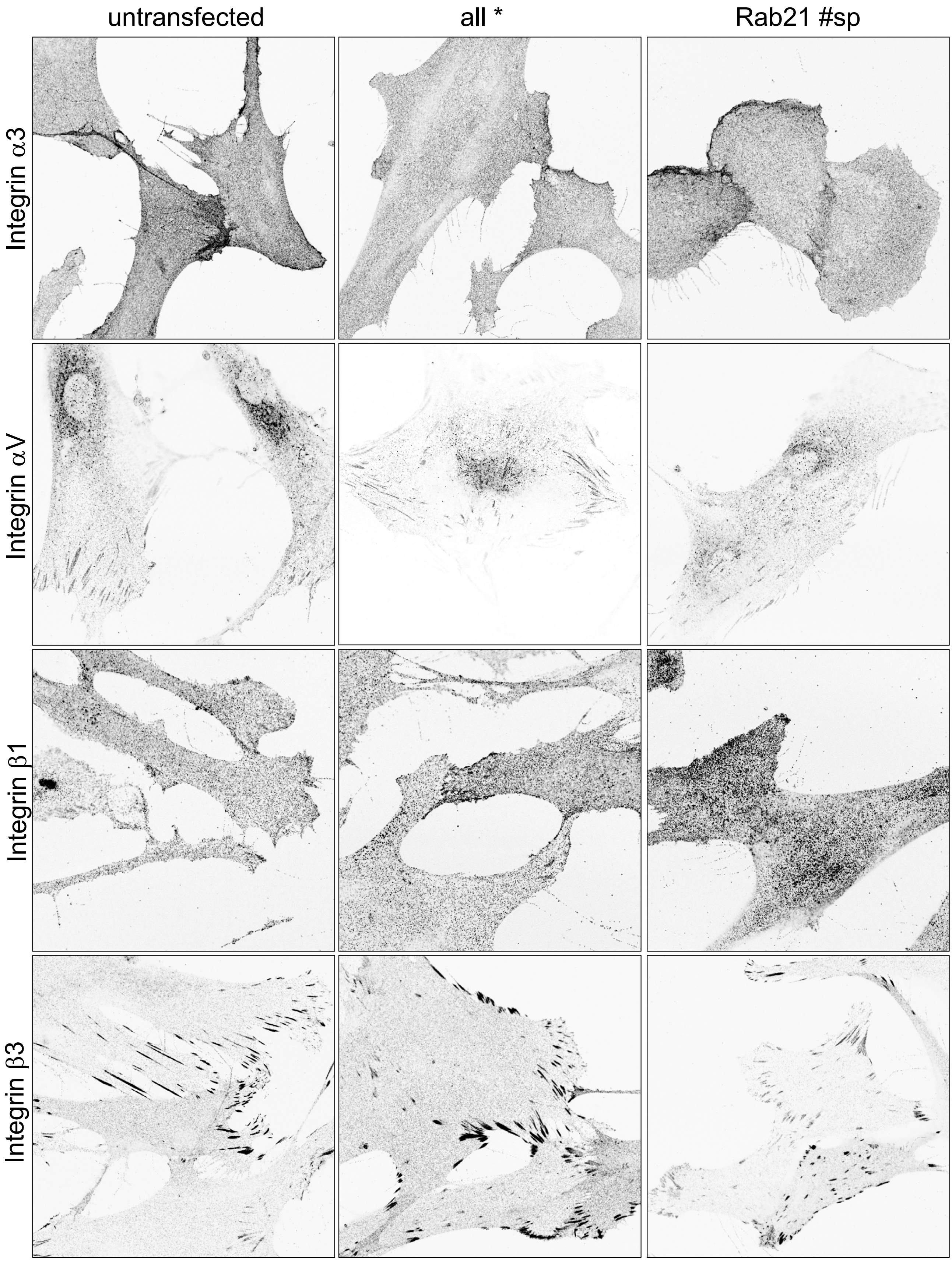

Supplementary Figure 5: The localization of integrins α3, αV, β1 and β3 is shown in untransfected, 'all *' and Rab21 smart pool siRNA transfected cells. Area 145x145μm is shown.

Supplementary Figure 6 Hooper et al.,

A

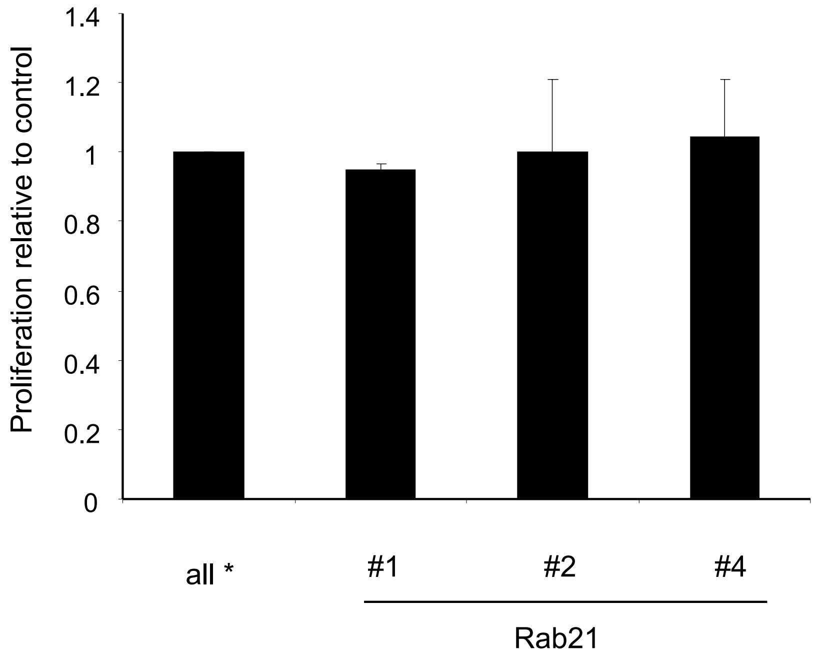

B

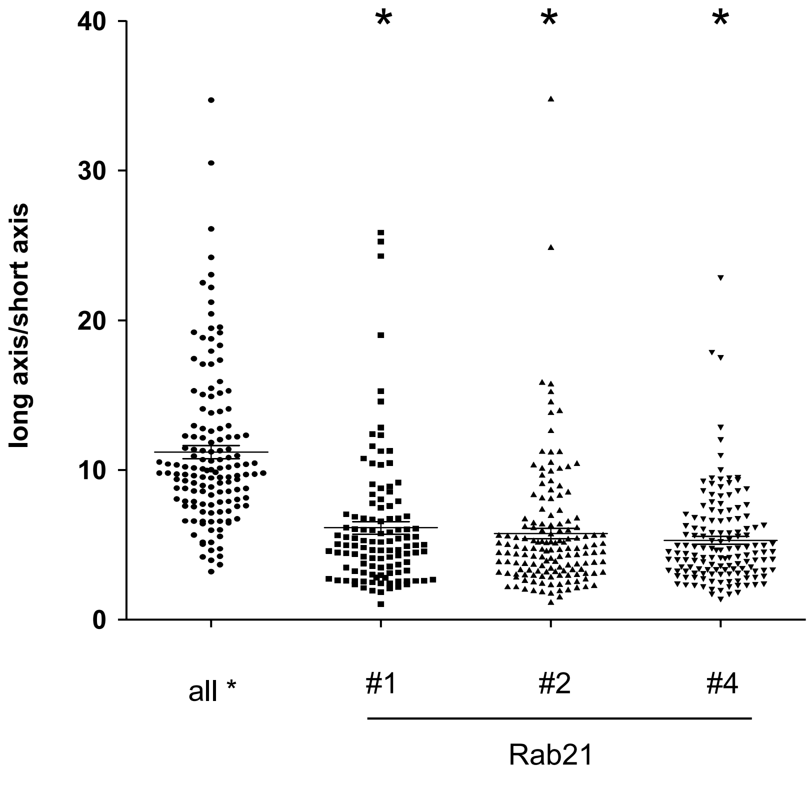

Supplementary Figure 6: A) The effect of Rab21 siRNA (oligos 1, 2, & 4) on CAF proliferation relative to allstars control siRNA is shown. B) The effect of Rab21 siRNA (oligos 1, 2, & 4) on CAF morphology compared to allstars control siRNA is shown. The long and short axis of >100 cells was measured for each condition (data pooled from two independent experiments, * indicates p<0.001 Student's t-test). The long axis/short axis value is shown for each cell.
