# Supplementary material for: A chemical biology screen reveals a role for Rab21-mediated control of actomyosin contractility in fibroblast-driven cancer invasion
Source: Br J Cancer. 2009 Dec 1;102(2):392–402. doi: 10.1038/sj.bjc.6605469 (PMC2816649; doi:10.1038/sj.bjc.6605469)
Supplement: Supplementary Table 1 [file 6605469x2.doc]

**Table 1**

The average normalized matrix contraction value is shown for each compound in the screen at both 1 and 10μM. Blank rows indicate no compound present in the well. ROCK inhibitors are highlighted in green. HMG CoA reductase inhibitors are highlighted in red. Broad spectrum MMP inhibitor is highlighted in turquoise. Cell cycle inhibitor and induces of apoptosis that ‘scored’ as a result of reducing overall cell numbers are highlighted in blue.

The chemical library for screening was in part made from assembling a range of inhibitors and growth factors known to affect signalling pathways into a 96 well plate and in part from a commercially available set of kinase inhibitors in 96 well format. The commercial sets of compounds was purchased from Merck (cat. no. #539744 & cat. no. #539745). The following reagents were purchased individually: Y27632 (Tocris #1254), H1152 (Merck #555550), GM6001 (Merck #364205), HA1077 (Tocris #541), SB431542 (Tocris #1614), UO126 (Promega #v1121), SP600125 (Tocris #1496), AG1478 (Sigma T-4182), K252a (Tocris #1683), LY294002 (Tocris #1130), Simvastatin (Merck #567021), Genstein (Tocris #1110), MG132 (Tocris #1748), Lovastatin (Merck #438186), Trichostatin A (Tocris #1406), Decitabine (Tocris #2624), TGFβ (Peprotech #100-21c), HGF (Biosource #PMG-0254), EGF (Sigma E-9644), SDF-1α (Sigma S-8032), Thrombin (Sigma T-4648), TPA (Sigma P-8139).

| **Normalized-score** | | **Compound** |
| --- | --- | --- |
| **10μM** | **1μM** |  |
| 0.99797 | 0.979264 | DMSO |
| 0.964562 | 0.915783 | AG 1024 |
| 1.148133 | 0.994763 | AGL 2043 |
| 1.02367 | 1.002649 | Akt Inhibitor IV |
| 0.2749 | 0.56518 | Akt Inhibitor V, Triciribine |
| 1.067567 | 0.964879 | Akt Inhibitor VIII, Isozyme-Selective, Akti-1/2 |
| 0.989066 | 0.989657 | Akt Inhibitor X |
| 0.297881 | 0.871522 | PDK1/Akt/Flt Dual Pathway Inhibitor |
| 1.098379 | 1.006641 | Aurora Kinase Inhibitor II |
| 1.302927 | 1.169698 | Bcr-abl Inhibitor |
| 0.832033 | 0.998838 | Bisindolylmaleimide I |
| 1.041121 | 0.975467 |  |
| 0.930377 | 0.946229 | DMSO |
| 1.018201 | 0.923958 | Bisindolylmaleimide IV |
| 1.242745 | 0.971197 | BPIQ-I |
| 1.092224 | 0.917928 | Chelerythrine Chloride |
| 1.049074 | 1.074015 | Compound 56 |
| 0.907815 | 0.909663 | DNA-PK Inhibitor II |
| 0.990201 | 0.976666 | DNA-PK Inhibitor III |
| 0.318112 | 0.500354 | PI-103 |
| 1.026093 | 1.036245 | Diacylglycerol Kinase Inhibitor II |
| 1.073905 | 1.06603 | DMBI |
| 0.973231 | 0.952358 | EGFR/ErbB-2 Inhibitor |
| 0.973946 | 0.952069 |  |
| 1.075817 | 1.002567 | DMSO |
| 0.450732 | 0.287247 | EGFR Inhibitor |
| 1.002724 | 0.987529 | EGFR/ErbB-2/ErbB-4 Inhibitor |
| 1.231357 | 1.133874 | Flt-3 Inhibitor |
| 0.96901 | 1.009891 | Flt-3 Inhibitor II |
| 1.040344 | 1.093126 | cFMS Receptor Tyrosine Kinase Inhibitor |
| 0.814941 | 1.112527 | Gš 6976 |
| 1.028363 | 0.97203 | Gš 6983 |
| 0.83051 | 1.020086 | GTP-14564 |
| 0.332096 | 0.916889 | Herbimycin A, Streptomyces sp. |
| 0.819244 | 0.881816 | Flt-3 Inhibitor III |
| 0.953061 | 0.913004 |  |
| 1.094943 | 1.002096 | DMSO |
| 1.03975 | 1.062607 | IGF-1R Inhibitor II |
| 1.174579 | 1.08228 | IRAK-1/4 Inhibitor |
| 0.328631 | 0.76618 | JAK Inhibitor I |
| 0.871063 | 1.066501 | JAK3 Inhibitor II |
| 0.939373 | 1.068356 | JAK3 Inhibitor IV |
| 0.707213 | 1.049517 | JAK3 Inhibitor VI |
| 0.981777 | 1.165235 | Lck Inhibitor |
| 0.853369 | 1.082181 | LY 294002 |
| 0.958857 | 0.998294 | LY 303511 |
| 1.102058 | 1.055926 | Met Kinase Inhibitor |
| 0.942961 | 1.012671 |  |
| 1.01127 | 1.029473 | DMSO |
| 1.038135 | 1.059168 | PD 158780 |
| 1.107374 | 1.200234 | PD 174265 |
| 1.001619 | 1.116338 | PDGF Receptor Tyrosine Kinase Inhibitor II |
| 0.860872 | 0.952267 | PDGF Receptor Tyrosine Kinase Inhibitor III |
| 0.351908 | 0.292353 | PDGF Receptor Tyrosine Kinase Inhibitor IV |
| 0.990803 | 0.937502 | PDGF RTK Inhibitor |
| 0.377844 | 0.950122 | PKR Inhibitor |
| 1.066585 | 1.014065 | PKR Inhibitor, Negative Control |
| 0.930019 | 1.0755 | PI 3-Kg Inhibitor |
| 0.986081 | 1.06316 | PI 3-KbInhibitor II |
| 0.947173 | 0.995967 |  |
| 1.075756 | 1.017958 | DMSO |
| 1.060575 | 0.94641 | PP3 |
| 1.217372 | 1.171843 | PP1 Analog II, 1NM-PP1 |
| 1.091775 | 1.145843 | PKCbII/EGFR Inhibitor |
| 0.91311 | 1.036055 | PKCb Inhibitor |
| 0.781502 | 0.854159 | Rapamycin |
| 1.041182 | 0.986407 | Rho Kinase Inhibitor III, Rockout |
| 0.339416 | 0.314904 | Rho Kinase Inhibitor IV |
| 0.295938 | 0.303769 | Staurosporine, N-benzoyl- |
| 0.926195 | 1.051282 | Src Kinase Inhibitor I |
| 0.958888 | 0.925633 | SU11652 |
| 0.942838 | 1.104171 |  |
| 0.960739 | 1.05666 | DMSO |
| 1.072321 | 1.171182 | Syk Inhibitor |
| 1.024927 | 1.0601 | Syk Inhibitor II |
| 1.023404 | 1.125519 | Syk Inhibitor III |
| 0.909011 | 0.906784 | TGF-b RI Kinase Inhibitor |
| 0.886 | 0.970075 | TGF-b RI Inhibitor III |
| 1.013928 | 1.05035 | AG 9 |
| 1.094372 | 1.026685 | AG 490 |
| 1.046979 | 1.069932 | AG 112 |
| 1.137972 | 1.119679 | AG 1295 |
| 1.066136 | 1.155593 | AG 1296 |
| 0.968447 | 1.043759 |  |
| 0.85295 | 0.966083 | DMSO |
| 0.975439 | 1.072893 | AG 1478 |
| 1.062609 | 1.093135 | VEGF Receptor 2 Kinase Inhibitor I |
| 0.983628 | 1.009421 | VEGF Receptor Tyrosine Kinase Inhibitor II |
| 0.963336 | 0.963385 | VEGF Receptor Tyrosine Kinase Inhibitor III, KRN633 |
| 1.045874 | 1.043388 | VEGF Receptor 2 Kinase Inhibitor II |
| 0.872578 | 0.976485 | VEGF Receptor 2 Kinase Inhibitor III |
| 1.134118 | 1.128394 | VEGF Receptor 2 Kinase Inhibitor IV |
| 1.045455 | 1.118557 | VEGF Receptor 3 Kinase Inhibitor, MAZ51 |
| 0.778455 | 1.094991 | Aurora Kinase Inhibitor III |
| 0.265188 | 0.291611 | Staurosporine, Streptomyces sp. |
| 0.992501 | 1.061667 |  |
| 0.893951 | 0.838988 | DMSO |
| 1.167466 | 0.974333 | KN-62 |
| 0.988223 | 0.975871 | ATM Kinase Inhibitor |
| 1.18917 | 0.953904 | ATM/ATR Kinase Inhibitor |
| 0.846072 | 1.072026 | Alsterpaullone |
| 0.367859 | 0.995013 | Alsterpaullone, 2-Cyanoethyl |
| 1.085816 | 0.914345 | Aloisine A |
| 1.010883 | 0.886939 | Aloisine, RP106 |
| 1.381567 | 0.740692 | Aminopurvalanol A |
| 1.168914 | 0.91944 | AMPK Inhibitor, Compound C |
| 0.813567 | 0.874275 | Aurora Kinase Inhibitor III |
| 0.90951 | 0.701897 |  |
| 1.121476 | 0.931459 | DMSO |
| 0.230202 | 0.913192 | Aurora Kinase/Cdk Inhibitor |
| 0.864169 | 1.074774 | Indirubin-3′-monoxime |
| 1.004235 | 1.041096 | BAY 11-7082 |
| 1.089355 | 1.02439 | Bohemine |
| 0.227713 | 0.813757 | Cdk1 Inhibitor |
| 1.000477 | 1.091213 | Cdk1 Inhibitor, CGP74514A |
| 0.134879 | 0.26012 | Cdk1/2 Inhibitor III |
| 1.009768 | 0.938584 | Cdk1/5 Inhibitor |
| 1.03459 | 1.027863 | Casein Kinase I Inhibitor, D4476 |
| 1.123993 | 1.03632 | Casein Kinase II Inhibitor III, TBCA |
| 0.987047 | 0.938404 |  |
| 1.058836 | 0.97584 | DMSO |
| 1.026609 | 1.114075 | Cdk4 Inhibitor |
| 1.14116 | 1.109057 | Cdk4 Inhibitor II, NSC 625987 |
| 1.120377 | 1.207319 | Cdk4 Inhibitor III |
| 1.119053 | 1.130463 | Cdc2-Like Kinase Inhibitor, TG003 |
| 1.425507 | 1.157923 | Chk2 Inhibitor II |
| 1.318482 | 1.068273 | Compound 52 |
| 0.96671 | 1.002372 | Cdk2 Inhibitor III |
| 0.179388 | 0.923394 | Cdk2 Inhibitor IV, NU6140 |
| 0.387925 | 0.190724 | Cdk/Crk Inhibitor |
| 1.164454 | 1.050023 | ERK Inhibitor III |
| 1.14116 | 0.979999 |  |
| 1.094414 | 1.152422 | DMSO |
| 0.210999 | 1.011509 | ROCK Inhibitor, Y-27632 |
| 1.131355 | 1.18193 | ERK Inhibitor II, FR180204 |
| 1.086101 | 1.176804 | ERK Inhibitor II, Negative control |
| 0.208805 | 0.984369 | Fascaplysin, Synthetic |
| 1.192515 | 1.137135 | GSK-3b Inhibitor I |
| 1.263387 | 1.09871 | GSK-3b Inhibitor II |
| 1.169295 | 1.061678 | GSK-3b Inhibitor VIII |
| 0.748971 | 0.736133 | GSK-3 Inhibitor IX |
| 1.068226 | 0.988291 | GSK-3 Inhibitor X |
| 1.016992 | 1.053923 | GSK-3b Inhibitor XI |
| 1.013358 | 0.920804 |  |
| 1.038134 | 1.074711 | DMSO |
| 1.304939 | 1.146627 | SU6656 |
| 0.859254 | 1.002759 | GSK-3 Inhibitor XIII |
| 0.964642 | 1.148178 | Isogranulatimide |
| 0.108438 | 0.866218 | IC261 |
| 0.998446 | 1.107724 | IKK-2 Inhibitor IV |
| 0.589275 | 0.777113 | Indirubin Derivative E804 |
| 1.096327 | 1.10354 | JNK Inhibitor II |
| 1.233555 | 1.105304 | JNK Inhibitor, Negative Control |
| 1.094979 | 0.994433 | JNK Inhibitor V |
| 0.751031 | 1.036333 | JNK Inhibitor IX |
| 0.894849 | 1.037875 |  |
| 0.978094 | 0.989936 | DMSO |
| 0.314968 | 0.215045 | MK2a Inhibitor |
| 1.11121 | 0.993292 | JNK Inhibitor VIII |
| 0.179747 | 0.951413 | K-252a, Nocardiopsis sp. |
| 1.18953 | 1.146903 | Kenpaullone |
| 1.162468 | 1.043287 | KN-93 |
| 1.090836 | 0.95995 | MEK Inhibitor I |
| 1.036652 | 1.10841 | MEK Inhibitor II |
| 1.317091 | 0.959217 | MEK1/2 Inhibitor |
| 1.016075 | 0.99012 | MNK1 Inhibitor |
| 1.418466 | 0.918499 | NF-kB Activation Inhibitor |
| 1.099802 | 0.924957 |  |
| 0.97794 | 0.968308 | DMSO |
| 1.268535 | 1.022893 | p38 MAP Kinase Inhibitor III |
| 1.185261 | 1.133764 | p38 MAP Kinase Inhibitor |
| 1.362757 | 1.006195 | PD 98059 |
| 1.488019 | 1.208645 | PD 169316 |
| 1.154823 | 1.039475 | SB220025 |
| 1.124679 | 0.951058 | Purvalanol A |
| 0.845428 | 1.125686 | GSK-3b Inhibitor XII, TWS119 |
| 0.923719 | 0.836797 | H-89, Dihydrochloride |
| 1.251499 | 0.9009 | SB 202474, Negative control for p38 MAPK inhibition studies |
| 1.097291 | 0.981129 | SB 202190 |
| 0.963435 | 0.915238 |  |
| 0.837673 | 1.06857 | DMSO |
| 1.348282 | 0.919945 | SB 203580 |
| 0.219359 | 0.847259 | HA 1077, Dihydrochloride |
| 0.843952 | 1.059694 | SB 218078 |
| 0.943342 | 0.958932 | SC-68376 |
| 1.35239 | 1.043208 | SKF-86002 |
| 0.992126 | 1.095771 | Sphingosine Kinase Inhibitor |
| 0.103857 | 0.145077 | Staurosporine, Streptomyces sp. |
| 0.831266 | 0.954669 | STO-609 |
| 1.003099 | 1.08208 | SU9516 |
| 1.02564 | 0.878457 | Tpl2 Kinase Inhibitor |
| 1.063737 | 1.04759 |  |
| 1.023704 | 1.004286 |  |
| 1.003954 | 0.955977 | DMSO |
| 0.772374 | 0.923663 | Y27632 |
| 0.07548 | 0.781902 | H1152 |
| 0.75124 | 0.77115 | GM6001 |
| 0.951402 | 0.953432 | SB431542 |
| 1.008013 | 1.032513 | UO126 |
| 1.058867 | 1.036604 | SP600125 |
| 1.000696 | 0.957376 | AG 1478 |
| 0.803406 | 0.941547 | LY294002 |
| 0.517657 | 0.878707 | Simvastatin |
| 1.028142 | 0.934572 | Genistein |
| 0.848279 | 0.703651 | MG132 |
| 0.622881 | 0.864281 | Lovastatin |
| 0.878874 | 0.95447 | Trichostatin A |
| 0.967026 | 0.997316 | Decitabin |
| 1.008328 | 1.089282 | Transforming Growth Factor beta 1 |
| 1.001151 | 0.926346 | Hepatocyte Growth Factor |
| 0.995315 | 1.050683 | Epidermal Growth Factor |
| 0.931862 | 1.009542 | SDF-1 |
| 1.053381 | 1.010677 | IFNgamma |
| 1.0142 | 1.017668 | Thrombin |
| 1.02567 | 1.014509 | SB 203580 |
| 0.988568 | 0.989923 |  |
| 0.871745 | 0.832875 | TPA |
| 0.960452 | 1.042144 | VEGF |
| 0.938937 | 1.006029 |  |
| 1.048498 | 0.991961 |  |
| 0.99601 | 1.044062 | DMSO |
